# Factors associated with initiation and persistence of urate-lowering therapy

**DOI:** 10.1186/s13075-016-1211-y

**Published:** 2017-01-17

**Authors:** Mats Dehlin, Emin Hoxha Ekström, Max Petzold, Ulf Strömberg, Gunilla Telg, Lennart T. H. Jacobsson

**Affiliations:** 1 Department of Rheumatology, University of Gothenburg, Gothenburg, Sweden; 2 Gothia Forum, Gothenburg, Sweden; 3 Swedish National Data Service, University of Gothenburg, Gothenburg, Sweden; 4 Health Metrics, University of Gothenburg, Gothenburg, Sweden; 5 AstraZeneca Nordic-Baltic, Södertälje, Sweden

**Keywords:** Gout, ULT, Persistence

## Abstract

**Background:**

Gout is the most common inflammatory arthritic disease and is caused by crystal deposition secondary to persistent hyperuricemia. Etiological treatment with urate-lowering therapy (ULT) has been available since the 1950s but previous studies have demonstrated suboptimal degree of treatment. In recent years we have seen recommendations for ULT earlier in the course of the disease, but there are few contemporary reports reflecting the current situation. Therefore we set out to investigate proportion receiving and persisting with ULT after gout diagnosis and predictors thereof.

**Method:**

A population-based cohort study using regional and national population-based registers. Cohort of patients (*n* = 7709) from western Sweden with incident gout aged 18 years and above from 2011 to 2013. An incident case of gout was defined as having been given a diagnosis of gout (ICD-10 M10, M14.0-14.1) not preceded by a gout diagnosis or a dispensation of ULT during the previous 5 years. Main outcome measures were cumulative incidence and predictors for start of, and persistence with, ULT in gout.

**Results:**

Within the first year after first gout diagnosis, 32% received ULT. Male sex, presence of diabetes or cardiovascular comorbidity, reduced kidney function but not diagnosed “end-stage kidney failure” increased the likelihood of receiving ULT. Of those starting ULT a majority (75%) did not persist with ULT treatment within the first 2 years. Age <50 years, lack of comorbidities, and “normal kidney function” or “end-stage kidney failure” were associated with non-persistence with ULT.

**Conclusions:**

Only a minority of patients received ULT and a majority of these did not persist with treatment over the next 2 years. However, the older patients with renal impairment and comorbidities, possibly suffering from a more severe gout disease, were more likely to receive and persist with treatment. There is thus still room for considerable improvement with regards to management of ULT in gout.

**Electronic supplementary material:**

The online version of this article (doi:10.1186/s13075-016-1211-y) contains supplementary material, which is available to authorized users.

## Background

Gout is the most common inflammatory arthritic disease and is caused by crystal deposition secondary to persistent hyperuricemia. It is also one of the few curable forms of arthritis. Gout is associated with higher age, male sex and several comorbidities, including renal dysfunction, metabolic syndrome, cardiovascular disease (CVD) and diuretic medication [1]. Gout has also in numerous studies been shown to predict mortality and CVD events [1], outcomes which have been suggested to be decreased with urate-lowering therapy (ULT) [2, 3]. Etiological treatment with ULT has been available since the 1950s [4] and previous studies from different countries have demonstrated suboptimal degree of treatment [5–8]. Furthermore, in recent years we have seen clinical guidelines with recommendations for ULT treatment earlier in the course of the disease [9], but there are few contemporary reports reflecting the current initiation and persistence with ULT and predictors thereof.

The aims of the present population-based cohort study were to determine: (1) the proportion of patients receiving ULT after gout diagnosis and predictors thereof, and (2) the proportion of patients persisting with ULT and predictors thereof.

## Methods

### Study design

This is a population-based cohort study of patients with newly diagnosed gout, investigating the treatment with ULT and predictors thereof using regional health care registers linked with several national mandatory population-based registers.

Ethical approval was granted by the Regional Ethics Committee in Gothenborg, Sweden. Informed consent from the patients was waived since the study only involved register linkage.

### Setting

Cases were identified from the health care register of all inhabitants in the Western Swedish Health Care Region (WSHCR), aged ≥ 18 years, from 1 January 2011 through 31 December 2013. The area represents approximately 20% of the total population of Sweden and is considered to be representative for Sweden as a whole with regard to health status and demographics [10, 11]. The Swedish health care system is tax-funded including private health care providers and all actors in this system report to the health registers.

### Data sources

The Western Swedish Health Care Register (VEGA) was used to identify incident cases with gout and their comorbidities. This register contains information about all in- and outpatient health care contacts in both specialized and primary care clinics, by both private and public care providers. The register contains date of contact and diagnosis given by the treating physician according to the Swedish version of the International Statistical Classification of Diseases (ICD), (the tenth version of ICD (ICD-10) has been used in Sweden since 1997).

The Swedish Prescribed Drug Register [12] contains information about all prescribed drugs dispensed by Swedish pharmacies since July 2005. This register was used to determine dispensation of ULT. The Anatomical Therapeutic Chemical Classification System (ATC codes) was used to identify the medical treatments.

Demographic data were obtained from Statistics Sweden [10], which holds data on residency as well as data on socioeconomic factors (e.g. marital status and level of formal education) for all persons residing in Sweden.

All blood test results for creatinine from 1 January 2010 to 31 December 2013 were identified for all cases in four regional laboratory databases.

### Study population of gout

**Fig. 1:**
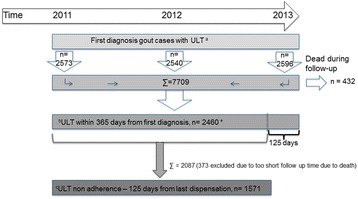
Flowchart of study population and outcomes. Incident cases of gout with ULT were identified from 2011-2013. ^a^An incident case of gout was defined as having been given a diagnosis of gout (ICD-10 M10, M14.0-14.1) at a visit in any care setting from 2011-01-01 to 2013-12-31 not preceded by a gout diagnosis or a dispensation of ULT during the previous 5 years. Out of these, the following two groups were identified: ^b^all cases with initiation of ULT within 365 days from diagnosis, ^c^all cases with ULT non adherence defined as not being dispensated new ULT125 days from last (ULT) dispensation. *For sensitivity analysis the group receiving ULT within 30 days from first diagnosis was identified

An incident case of gout was defined as having been given a diagnosis of gout (ICD-10 M10, M14.0-14.1) at a visit in any care setting from 1 January 2011 to 31 December 2013 not preceded by a gout diagnosis or a dispensation of ULT during the preceding 5-year period, see Fig. 1 for more information.

## Exposures

### Demographics

Level of formal education was divided into three groups: 9 school years or less, 10 to 12 years and more than 12 years.

### Comorbidities

Diabetes, common cardiovascular comorbidities (ischemic heart disease, arrhythmias, heart failure, peripheral vascular disease, cerebrovascular disease), renal disease and creatinine values were identified for all cases at the date of first gout diagnosis, date of first ULT dispensation and date of ULT stop. Comorbidities were defined as the presence of at least one prior visit to a physician in any care setting or hospitalization with a corresponding ICD-10-coded diagnosis (for ICD10 codes see Additional file 1: Table S1). A metabolic cardiovascular comorbidity index (MCCI) was computed, where the following conditions each rendered one point giving a maximum of six points: ischemic heart disease, arrhythmias, heart failure, peripheral vascular disease, cerebrovascular disease and diabetes (for ICD10 codes and details see Additional file 2: Table S2). Estimated glomerular filtration rate (eGFR) was calculated according to the Chronic Kidney Disease Epidemiology Collaboration (CKD-EPI) [13] using the creatinine value closest to the date of first gout diagnosis within 1 year before until 1 month after that date. Furthermore, we made the assumption that none of the cases were of African-American origin, since these, according to population statistics, represent less than 0.1% of the population. eGFR were divided into four ranges: “normal kidney function” defined as eGFR >60 mL/min/1.73 m^2^, “reduced kidney function” 60–31 mL/min/1.73 m^2^, “severely reduced kidney function” 30–10 mL/min/1.73 m^2^ and <10 mL/min/1.73 m^2^ “end-stage kidney failure”.

## Outcomes

Among all the incident gout cases identified from 2011 to 2013 two binary outcomes were defined. First, all incident cases with initiation of ULT within 365 days from diagnosis. Second, of those starting ULT, whom had not persisted with therapy, defined as no dispensation ≥125 days from the last dispensation. The choice of 125 days was based on the Swedish Pharmaceutical Benefit Scheme, which reimburse a maximum of 90 days drug supply at one purchase occasion [14]. In practice, packages for 100 days are often dispensed because of package sizes of 98 or 100 units. Thus, allowing for at least 80% adherence, one filled prescription would last for a maximum of 125 days. For sensitivity analysis, all incident cases with initiation of ULT within 30 days from diagnosis were identified. For further information, see Fig. 1.

Dispensation of ULT prescriptions included allopurinol (M04AA01), febuxostat (M04AA03), and probenecid (M04AB01).

### Statistical analysis

Descriptive statistics were used to summarize the demographic characteristics. Predictors (sex, age, level of education, comorbidity, renal disease, kidney function and year of gout diagnosis) for initiation of ULT within 365 days from diagnosis were assessed with Cox regression analyses. The proportional hazard assumption was visually evaluated and found valid in all included analyses. In addition, a sensitivity analyses was performed with all incident cases with initiation of ULT within 30 days from diagnosis to evaluate if point estimates were similar as in the analyses with 365 days of follow-up. Hazard ratios (HRs) with 95% confidence intervals (CIs) were calculated. Predictors for the likelihood of not adhering to ULT after 125 days were assessed with logistic regression models and presented as odds ratios (ORs) with 95% confidence intervals (CIs). All statistical analyses were performed using SAS 9.4 (SAS Institute Inc., Cary, NC, USA).

## Results

In total, 7709 incident cases of gout from 2011 to 2013 were identified and included. Of these, 68% were male, average age was 66 years and more than a third (35%) had low level of education, ≤ 9 years, see Table 1 for details. Less than half of the cases (47%), had diabetes or cardiovascular comorbidities and a diagnosed renal disease was present in 12% although decrease in kidney function defined as eGFR <60 mL/min/1.73 m^2^ was present in as many as 37% (Table 1) and Additional file 3: Table S3.

**Table 1:** Baseline variables at time of gout diagnosis, ULT start and for ULT stop

	At gout diagnosis	At ULT start	^a^At ULT stop
n	7709	2460	1571
Male sex, %	68.4	71.9	71.3
Age, mean	66.5	68.2	68.2
Level of education^b^: 9 years or less, %	35.1	37.5	38.2
Level of education: 10–12 years, %	41.8	41.8	42.5
Level of education: more than 12 years, %	19.2	17.2	16.4
Metabolic cardiovascular comorbidity index (MCCI)^c^, %			
0	53.1	42.5	43.4
1–2	34.9	41.0	39.5
3–4	11.4	15.6	16.0
5–6	0.6	0.9	1.2
>6	0.00	0.00	0.00
Renal disease^d^, %	12.1	17.8	18.8
”Normal kidney function”^e^, %	62.9	50.4	53.2
“Reduced kidney function”^f^, %	31.1	41.9	38.6
^“^Severely reduced kidney function”^g^, %	5.7	7.5	7.8
^“^End-stage kidney failure”^h^, %	0.3	0.2	0.4
2011,%	33.4	26.1	13.3
2012, %	33.0	35.8	36.4
2013, %	33.7	38.1	50.4

*ULT* urate-lowering treatment

^a^Defined as no ULT dispensation ≥125 days since last dispensation

^b^Missing data for level of education: 3.8% at gout diagnosis, 3.5% at ULT start and 2.9% at ULT stop

^c^For definition (ICD-10 codes) of metabolic cardiovascular comorbidity index see Additional file 2: Table S2

^d^For definition (ICD-10 codes) of renal disease see Additional file 1: Table S1

^e^Defined as eGFR >60 mL/min/1.73 m2

^f^Defined as eGFR 60–31 mL/min/1.73 m2

^g^Defined as eGFR 30–10 mL/min/1.73 m2

^h^Defined as eGFR <10 mL/min/1.73 m2

^e-h^Creatinine value closest to date of first gout diagnosis within 1 year before until 1 month after, a third (2555) of the incident cases did not have a creatinine value within 1 year before and 1 month after. The vast majority of these, 95%, did not have renal disease. For more details, see Additional file 3: Table S3.

### The proportion of patients receiving ULT after gout diagnosis and predictors thereof

**Fig. 2:**
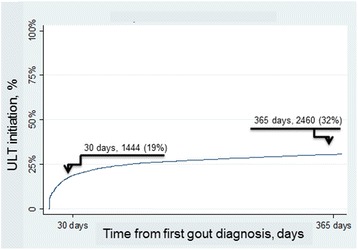
Proportion of patients receiving ULT within 30 and 365 days from diagnosis respectively, in incident patients with gout

Of the 7709 incident cases, 1444 (19%) and 2460 (32%) received ULT from the date of first gout diagnosis up through 30 and 365 days post-diagnosis respectively, see Fig. 2. The vast majority was prescribed allopurinol, less than 2% received probenecid and febuxostat was not used at all.

Male sex, age ≥ 60, “reduced kidney function” but not “end-stage kidney failure” by eGFR, presence of diagnosed renal disease and presence of ≥ 1 comorbidity all significantly (*p* < 0.05) predicted receiving ULT within 365 days by univariate COX regression (Table 2). Two different multivariate analyses were performed, one with renal disease defined by ICD10 coding and the other with renal function by eGFR as a proxy for renal pathology. Male sex and presence of ≥ 1 comorbidities both significantly (*p* < 0.05) and positively predicted start of ULT within 365 days in both models (Table 2). Renal disease also predicted start of ULT. When using eGFR as a proxy for renal pathology “reduced kidney function” but not “end-stage kidney failure” predicted start of ULT (Table 2). Age 70–79 positively predicted start of ULT when renal disease was defined by ICD10 coding (Table 2). The effects of level of education disappeared when adjusting for age and were therefore not included in the multivariate analyses. There was no significant effect of year of gout diagnosis, which suggests no major changes in treatment patterns during the study period.

**Table 2:** Predictors for first ULT dispensation within 365 days after diagnosis

		Predictors for ULT dispensation within 365 days (COX)		
	% with event	Univariate hazard ratio 95% CI	Multivariate* hazard ratio 95% CI (with eGFR)	Multivariate** hazard ratio 95% CI (with renal disease)
Male (ref)	30.33			
Female	26.39	0.87 (0.79–0.95)	0.87 (0.79–0.97)	0.86(0.78–0 .94)
Age, years				
20–49 (ref)	22.84			
50–59	23.21	1.03 (0.87–1.22)	0.90 (0.72–1.12)	0.96 (0.81–1.14)
60–69	30.14	1.38 (1.19–1.59)	0.90 (0.74–1.10)	1.15 (0.99–1.34)
70–79	34.19	1.61 (1.40–1.86)	0.82 (0.67–1.00)	1.25 (1.07–1.46)
80–	30.74	1.46 (1.26–1.69)	0.57 (0.46–0.71)	1.03 (0.87–1.21)
Level of education				
≤9 years (ref)	31.10			
10–12 years	28.78	0.92(0.83–1.31)		
>12 years	26.32	0.83(0.73–0.93)		
MCCI				
0 (ref)	23.53			
1–2	34.20	1.57 (1.44–1.72)	1.29 (1.15–1.45)	1.45 (1.31–1.60)
>2	38.79	1.91 (1.69–2.15)	1.43 (1.23–1.66)	1.73 (1.51–1.99)
Renal disease				
0 (ref)	27.48			
1	40.75	1.67 (1.50–1.87)		1.47 (1.31–1.64)
eGFR >60 mL/min/1.73 m^2^ “normal kidney function” (ref)	26.56			
eGFR 60–31 mL/min/1.73 m^2^ “reduced kidney function”	44.33	1.91 (1.73–2.11)	2.11 (1.88–2.37)	
eGFR 30–10 mL/min/1.73 m^2^^“^severely reduced kidney function”	44.71	2.01 (1.67–2.41)	2.31 (1.90–2.82)	
eGFR <10 mL/min/1.73 m^2^ “end-stage kidney failure”	30.77	1.14 (0.43–3.04)	1.17 (0.44–3.12)	
2011, ref				
2012		1.10 (0.99–1.21)		
2013		1.02 (0.92–1.13)		

*ULT* urate-lowering treatment, *CI* confidence interval, *eGFR* estimated glomerular filtration rate, *MCCI* metabolic cardiovascular comorbidity index

^*^Adjusted for sex, age, comorbidities and eGFR

^**^Adjusted for sex, age, comorbidities and renal disease defined by ICD 10 coding

A sensitivity analysis evaluating predictors for initiation of ULT up through 30 days after diagnosis of gout showed similar point estimates (Additional file 4: Table S4).

### The proportion of patients not persisting with ULT and predictors thereof

Of the 7709 incident cases, 2087 (27%) were followed up after their first ULT dispensation, a start of therapy which did not necessarily have to be within 365 days after diagnosis. Of these, 1571 (75%) did not persist with ULT treatment. The risk for not persisting with ULT was significantly (*p* < 0.05) higher for those with age < 50 years, total lack of comorbidities, no renal disease and “normal kidney function” by eGFR (Table 3), with similar point estimates in models using alternatively ICD coding or eGFR as a proxy for renal pathology.

**Table 3:** Predictors for not adhering to ULT

		Predictors for not persisting with ULT after 125 days^a^ (Log)		
	% with event	Univariate odds ratio 95% CI	Multivariate^b^ odds ratio 95% CI (with eGFR)	Multivariate^c^ odds ratio 95% CI (with renal disease)
Male (ref)	37.47			
Female	36.30	0.95 (0.78–1.16)	0.89 (0.71–1.13)	0.92 (0.75–1.13)
Age, years				
20–49 (ref)	54.29			
50–59	37.65	0.51 (0.36–0.73)	0.50 (0.31–0.79)	0.54 (0.38–0.77)
60–69	37.31	0.50 (0.37–0.68)	0.63 (0.42–0.95)	0.58 (0.42–0.80)
70–79	33.28	0.42 (0.31–0.57)	0.59 (0.39–0.91)	0.51 (0.37–0.71)
80–	32.55	0.41 (0.30–0.56)	0.70 (0.44–1.11)	0.52 (0.36–0.74)
MCCI				
0 (ref)	43.65			
1–2	32.67	0.63 (0.51–0.76)	0.75 (0.59–0.96)	0.74 (0.60–0.92)
>2	30.16	0.56 (0.42–0.73)	0.71 (0.52–0.99)	0.69 (0.51–0.93)
Renal disease				
0 (ref)	38.54			
1	29.57	0.67 (0.52–0.86)	0.74 (0.57–0.97)	
eGFR >60 mL/min/1.73 m^2^ “normal kidney function” (ref)	41.62			
eGFR 60–31 mL/min/1.73 m^2^ “reduced kidney function”	31.19	0.64 (0.51–0.79)		0.68 (0.53–0.87)
eGFR 30–10 mL/min/1.73 m^2^^“^severely reduced kidney function”	28.57	0.56 (0.37–0.85)		0.59 (0.37–0.92)
eGFR <10 mL/min/1.73 m^2^ “end-stage kidney failure”	25.00	0.47 (0.05–4.51)		0.53 (0.05–5.11)
2011, ref	37.40			
2012	33.87	0.86 (0.69–1.06)		
2013	41.62	1.19 (0.95–1.50)		

*ULT* urate-lowering treatment, *CI* confidence interval, *eGFR* estimated glomerular filtration rate, *MCCI* metabolic cardiovascular comorbidity index

^a^Defined as no ULT dispensation 125 days after last dispensation

^b^Adjusted for sex, age, comorbidities, and eGFR

^c^Adjusted for sex, age, comorbidities and renal disease

There was no significant effect of year for gout diagnosis, which suggests no major change in treatment patterns during the study period.

## Discussion

We identified 7709 incident cases of gout in the WSHCR, aged ≥ 18 years from 1 January 2011 through 31 December 2013. Of these, only 32% received ULT within 365 days after first gout diagnosis. Positive predictors thereof were presence of diabetes or cardiovascular comorbidity, renal disease, and “reduced kidney function” but not “end-stage kidney failure”. A positive predictive effect of age was only seen in one group, 70–79 years, and only in the multivariate model adjusted for renal disease. The vast majority did not persist with ULT and risks for not persisting with ULT were higher for those with age <50 years, total lack of comorbidities, no renal disease and “normal kidney function” by eGFR.

There are several possible limitations to our study. First, our case definitions were based on diagnoses of gout made in the clinical situation rather than according to the different proposed classification criteria [15–20], which may have led to misclassification bias. Previous studies by us in this setting have demonstrated a high validity for a more strict definition of gout requiring two or more visits with a gout diagnosis in primary care [21]. However, the comorbidity pattern is similar between the more strict definition and the one used in the present study [22] suggesting similar validity for the two definitions. Second, gout has an intermittent course with possibly long clinically silent phases, which may hamper any attempt to identify true incident cases of the disease. Third, there may be more factors such as other medications and comorbidities not covered in our analysis which may affect the initiation of ULT. Fourth, we had no specific data to elucidate to what extent non-persistance was explained by factors such as perceived lack of efficacy, side effects experienced, or simply patient or practitioner preference.

There are also several strengths of the present study. First, using the mandatory national health registers in Sweden, with a virtually complete coverage on an individual level, makes the results population representative. Second, loss to follow-up is not a problem since the Swedish population is possible to follow to death through the central statistics in Sweden. Third, the estimates for ULT treatment, laboratory data and socioeconomic data were retrieved from independent data sources.

Predictors for initiation of ULT in gout is not a well-studied topic, maybe reflecting some uncertainty of when to start treatment. Kuo et al. [23] showed in a study about eligibility for ULT in incident gout that the cumulative probability of fulfilling any treatment indication for ULT was 44% at diagnosis and 61% after 1 year. They also reported findings similar to ours with sex, comorbidity and kidney disease being predictors for initiation of ULT. Furthermore, persistence in our study was negatively affected by age < 50 years, which is in accordance to a historical study (by Horsburgh et al. [24]) on allopurinol prescription and persistence in New Zealand 2005/06.

Predictors of persistence with ULT was also investigated by Harrold et al. [25] in 4166 patients with gout in the US from 2000 to 2006, reporting age <50 and fewer comorbidities to predict worse persistence, results that are similar to our findings with lack of comorbidities significantly predicting low persistence. “Normal renal function” (eGFR > 60) or “end-stage renal failure” (<10 mL/min/1.73 m^2)^ respectively predicted worse persistence with ULT in the present study. Interestingly, Sarawate et al. [26], in a managed care database study from 2000 to 2002 in the US also found that presence of renal impairment negatively affected persistence with ULT. However, the renal impairment in their study was based on diagnosis rather than eGFR hampering exact comparison. Finally, Briesacher et al. [27] showed in a study from 2008 that gout had by far the poorest persistence with therapy when compared to six other chronic diseases, where only 37% of patients with gout achieved at least 80% persistence with ULT. This study also showed that increasing age and presence of comorbidities improved persistence for all studied diseases, including gout. Compared to all these studies, our results are based on more contemporary data and suggest that low levels of initiation and persistence of ULT continues to be a health care problem. Furthermore, we could not demonstrate any change in treatment pattern during our study period.

Some of the negative prognostic markers favoring ULT initiation, highlighted in the proposed EULAR guidelines from 2016 [9], have already been acted upon by the clinicians in our study, such as the presence of comorbidities and renal impairment while others, such as age below 40, on the contrary did not predict ULT initiation. There are still considerable gaps of knowledge when initiation of ULT is appropriate after first diagnosis of gout. On the other hand, our results clearly show that the proportion of patients receiving ULT in diagnosed gout continuous to be low. In the present study, we have focused on patient characteristics affecting ULT, which of course is not the only explanation. Different aspects of clinical inertia come to play here. The physicians knowledge and attitude to guidelines is a problem well observed and might be improved by shared decision making and enhanced guideline development and dissemination [28]. Furthermore, consultation patterns with patients not consulting for repeated attacks when having acute anti-inflammatory treatment at hand thus not giving the physician the opportunity to discuss ULT, awareness of patient attitudes, knowledge and preferences towards gout and ULT are important factors to consider in the strive for improvement [29, 30].

## Conclusions

In the present study, we demonstrate that initiation and persistence to ULT continues to be poor, although predictors thereof indicate better results for those with a stronger indication for therapy. The long-term consequences of poor ULT treatment of gout need to be exactly determined. Nevertheless, future research should also aim at identifying and addressing the barriers to starting and persisting with ULT over time and to overcome these.

## Additional files

Additional file 1: Table S1. Definition of comorbidities by ICD10 codes and ULT by ATC codes. (DOCX 91 kb)
Additional file 2: Table S2. Metabolic cardiovascular comorbidity index (MCCI). (DOCX 91 kb)
Additional file 3: Table S3. Distribution of kidney function defined by eGFR and presence of renal disease in incident cases at date of first gout diagnosis and missing. (DOCX 92 kb)
Additional file 4: Table S4. Predictors for first ULT dispensation within 30 days after diagnosis. (DOCX 93 kb)

## Abbreviations

ATC: anatomical therapeutic chemical classification system
CIs: confidence intervals
CKD-EPI: Chronic Kidney Disease Epidemiology Collaboration
CVD: cardiovascular disease
eGFR: estimated glomerular filtration rate
HRs: hazard ratios
ICD: International Statistical Classification of Diseases
MCCI: metabolic cardiovascular comorbidity index
ORs: odds ratios
ULT: urate-lowering therapy
VEGA: Western Swedish Health Care Register
WSHCR: Western Swedish Health Care Region
