# Supplementary material for: Factors associated with initiation and persistence of urate-lowering therapy
Source: Arthritis Res Ther. 2017 Jan 17;19:6. doi: 10.1186/s13075-016-1211-y (PMC5240247; doi:10.1186/s13075-016-1211-y)
Supplement: Additional file 1: Table S1. — Definition of comorbidities by ICD10 codes and ULT by ATC codes. (DOCX 91 kb) [file 13075_2016_1211_MOESM1_ESM.docx]

| Co-morbidity | ICD-10 code | ATC-code |
| --- | --- | --- |
| Hypertension | I10-15 |  |
| Diabetes | E10-14, O24 |  |
| Ischemic Heart Disease | I20-25 |  |
| Heart failure | I 50 |  |
| Stroke | I60-64, G45 |  |
| Renal disease | N00-08  N11-22 |  |
| ULT | | |
| Allopurinol |  | M04AA01 |
| Probenecid |  | M04AB01 |
| Febuxostat |  | M04AA03 |

Supplementary Table 1 – Definition of co-morbidities by ICD10-codes and ULT by ATC-codes.
