# Supplementary material for: Factors associated with initiation and persistence of urate-lowering therapy
Source: Arthritis Res Ther. 2017 Jan 17;19:6. doi: 10.1186/s13075-016-1211-y (PMC5240247; doi:10.1186/s13075-016-1211-y)
Supplement: Additional file 2: Table S2. — Metabolic cardiovascular comorbidity index (MCCI). (DOCX 91 kb) [file 13075_2016_1211_MOESM2_ESM.docx]

| Metabolic cardiovascular comorbidity index (MCCI), max 6 points | | |
| --- | --- | --- |
| Assigned weight for diseases | Conditions | ICD-10 code |
| 1 | Ischemic heart disease | I 20-25 |
| 1 | Arrhythmias | I 44-49 |
| 1 | Congestive heart failure | I 50 |
| 1 | Cerebrovascular disease | I 60-64, G45 |
| 1 | Peripheral vascular disease | I 70-71 |
| 1 | Diabetes | E 10-14 |

Supplementary Table 2 Metabolic cardiovascular comorbidity index (MCCI), max 6 points and 1 point for each group of conditions
