# Supplementary material for: Factors associated with initiation and persistence of urate-lowering therapy
Source: Arthritis Res Ther. 2017 Jan 17;19:6. doi: 10.1186/s13075-016-1211-y (PMC5240247; doi:10.1186/s13075-016-1211-y)
Supplement: Additional file 3: Table S3. — Distribution of kidney function defined by eGFR and presence of renal disease in incident cases at date of first gout diagnosis and missing. (DOCX 92 kb) [file 13075_2016_1211_MOESM3_ESM.docx]

| Renal function | eGFR >60 mL/min/1.73m^2^  ”normal kidney function” (n) | % | eGFR 60 - 31 mL/min/1.73m^2^  “reduced kidney function” (n) | % | eGFR 30-10 mL/min/1.73m^2 “^severely reduced kidney function” (n) | % | eGFR <10 mL/min/1.73m^2 ”^normal kidney failure” (n) | % | Missing | % | Total |
| --- | --- | --- | --- | --- | --- | --- | --- | --- | --- | --- | --- |
| Renal disease |  |  |  |  |  |  |  |  |  |  |  |
| 0 | 3 026 | 93.34 | 1 227 | 76.40 | 104 | 35.49 | 2 | 15.38 | 2420 | 94.72 | 6779 |
| 1 | 216 | 6.66 | 379 | 23.60 | 189 | 64.51 | 11 | 84.62 | 135 | 5.28 | 930 |

Supplementary table 3 Distribution of kidney function defined by eGFR and presence of renal disease in incident cases at date of first gout diagnosis and missing
