# Supplementary material for: Factors associated with initiation and persistence of urate-lowering therapy
Source: Arthritis Res Ther. 2017 Jan 17;19:6. doi: 10.1186/s13075-016-1211-y (PMC5240247; doi:10.1186/s13075-016-1211-y)
Supplement: Additional file 4: Table S4. — Predictors for first ULT dispensation within 30 days after diagnosis. (DOCX 93 kb) [file 13075_2016_1211_MOESM4_ESM.docx]

|  | Predictors for ULT dispensation within 30 days | | |
| --- | --- | --- | --- |
|  | Univariate Hazard ratio 95% CI | Multivariate* Hazard ratio 95% CI (with eGFR) | Multivariate** Hazard ratio 95% CI (with renal disease) |
| Sex, (ref male) | 0.92(0.82-1.02) | 0.90(0.79-1.02) | 0.90(0.80-1.01) |
| Age, years |  |  |  |
| 20-49 (ref) |  |  |  |
| 50-59 | 1.10(0.89-1.36) | 0.88(0.67-1.16) | 1.03(0.83-1.27) |
| 60-69 | 1.37(1.14-1.65) | 0.86(0.67-1.09) | 1.14(0.94-1.38) |
| 70-79 | 1.63(1.37-1.95) | 0.79(0.61-1.01) | 1.24(1.02-1.51) |
| 80- | 1.53(1.28-1.85) | 0.58(0.44-0.76) | 1.06(0.86-1.31) |
| MCCI |  |  |  |
| 0 (ref) |  |  |  |
| 1-2 | 1.57(1.41-1.76) | 1.29(1.12-1.49) | 1.45(1.28-1.65) |
| >2 | 1.93(1.67-2.24) | 1.51(1.25-1.81) | 1.74(1.47-2.07) |
| Renal disease |  |  |  |
| 0 (ref) |  |  |  |
| 1 | 1.66(1.45-1.90) |  | 1.45(1.26-1.67) |
| eGFR >60 mL/min/1.73m^2^  ”normal kidney function” (ref) |  |  |  |
| eGFR 60 - 31 mL/min/1.73m^2^  “reduced kidney function” | 1.78(1.57-2.01) | 1.91(1.65-2.20) |  |
| eGFR 30-10 mL/min/1.73m^2 “^severely reduced kidney function” | 1.93(1.54-2.41) | 2.13(1.68-2.70) |  |
| eGFR <10 mL/min/1.73m^2 ”^endstage kidney failure” | 0.85(0.21-3.39) | 0.86(0.21-3.43) |  |
| 2011, ref |  |  |  |
| 2012 | 1.09(0.96-1.24) |  |  |
| 2013 | 0.99(0.87-1.13) |  |  |

Supplementary table 4 Predictors for first ULT dispensation within 30 days after diagnosis, *adjusted for sex, age, comorbidities, and eGFR ** Adjusted for sex, age, comorbidities and renal disease
